# Automating the initial physics chart‐checking process

**DOI:** 10.1120/jacmp.v10i1.2855

**Published:** 2009-02-11

**Authors:** Eli E. Furhang, James Dolan, Jussi K. Sillanpaa, Louis B. Harrison

**Affiliations:** ^1^ Department of Radiation Oncology Beth Israel Medical Center 10 Union Square East New York NY U.S.A.

**Keywords:** statistical process control, chart review, treatment planning quality assurance

## Abstract

The initial physics chart check, an essential quality assurance process, verifies that the physician intent is properly expressed in the treatment plan, the treatment plan is reasonable, and the Record and Verify (RV) system properly captures the plan parameters. In this work the process was automated by characterizing the initial physics chart check as a universal set of steps, compartmentalized into intra‐plan and inter‐plan reviews. The intra‐plan review confirms the diagnosis‐prescription‐plan correlation, and verifies transfer accuracy of the signed treatment plan parameters into the RV system. The inter‐plan review tabulates all RV parameters for similar cases, and highlights outliers. The tabulation of RV parameters for similar cases enables a summation of experience across staff members, and facilitates a comparison using the Statistical Process Control (SPC) formalism. A summary sheet, added to each reviewed chart, automatically documents deviations noted during the review process. Forty‐five patient charts were analyzed using the software. The length of time for the entire initial chart‐checking process was reduced from about an hour to a few minutes. The code developed in this work allows the user to consider the big picture, trusting the software to track details.

PACS number 89.20.Bb

## I. INTRODUCTION

Regulatory standards, as well as AAPM recommendations, require treatment courses be reviewed before 10% of the dose is delivered.^(1)^ At Beth Israel Medical Center, patient treatment courses are reviewed after the plan is approved by the physician but before treatment commences. The manuscript will focus on this pre‐treatment physics chart‐checking process. The patient treatment course is also reviewed after the first treatment fraction is delivered and on a weekly basis thereafter, but these processes are outside the scope of this manuscript.

Generally, the initial physics chart check confirms correlation between diagnosis, prescription, and plan, and ensures accurate information transfer between the plan and the RV system. The process also reviews consistency and sensibility, which relies on an experienced physicist's judgment.

A thorough initial physics check requires a review of the diagnosis, prescription (modality, isodose, daily dose, number of fractions to be treated, and special instructions), DVH parameters, and physician approval, as well as about 20 field‐specific parameters. Typically, breast plans have 2 fields, while head and neck plans with split fields could have about 20 fields. Consequently, breast plans would entail reviewing about 50 parameters, while a head and neck would involve approximately 400 parameters. Each of these parameters could have a critical impact on the patient's outcome. Each pre‐treatment review could last an hour and, in a busy clinical program, it is not unusual for the checker to review several charts sequentially. Given the number of parameters and charts, it is conceivable that even a conscientious and experienced checker might miss a detail. The purpose of this work was to formalize the pre‐treatment chart review procedure, and automate it as much as possible, while maintaining the quality of the process.

## II. METHODS

Once a treatment plan is approved, it is electronically transferred to the RV system (MultiAccess, IMPAC Medical Systems, Mountain View, CA). The fields are then modified manually by the therapists and physicists to incorporate additional information such as couch coordinates, field sequencing, and dose rates. Therefore, it is best to extract patient treatment information only after all such manual entries occur. Extracting the plan data from the RV system also avoids the need for multiple interfaces for each of our 3 external beam and radiosurgery treatment planning software vendors. A report (Crystal Reports, Seagate Software Inc, San Jose, CA) was developed to extract the diagnosis, prescription, and plan parameters from the RV system into an Excel (Microsoft Corporation, Redmond, WA) spreadsheet. This report is also designed to replicate the external beam treatment planning system printout, for visual comparison. Although developed for specific treatment planning and RV vendors, this interface can be adapted to other vendors as needed.

An Excel macro was written in Visual Basic (Microsoft Corporation, Redmond, WA) to guide the user through the review process and to automatically review the data when possible. The initial physics chart‐checking process was divided into intra‐plan and inter‐plan reviews. The macro performs both reviews sequentially, as described below.

### A. Intra‐plan Review

**Figure 1:**
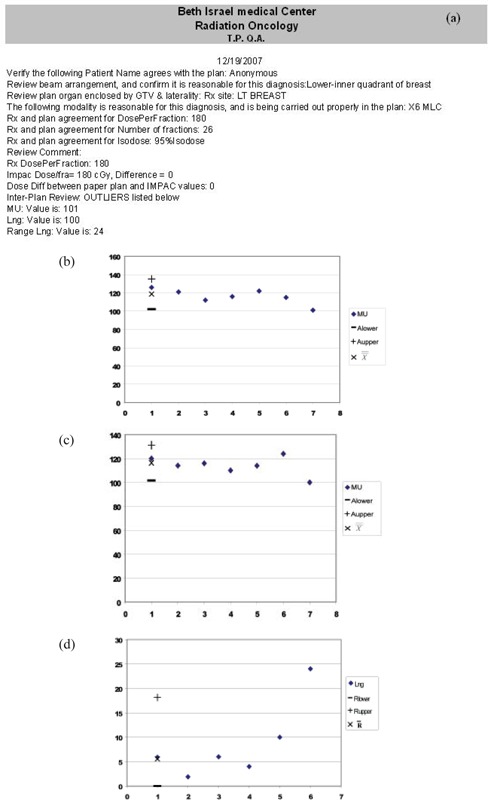
a typical printout for a breast patient. Three inter‐plan outliers are noted, as shown in Figs. (b), (c), and (d).

The intra‐plan review confirms diagnosis‐prescription‐plan correlation, the accuracy of transfer of plan parameters, and plan parameters self‐consistency. The review consists of a series of prompts that guides the user to evaluate the correlation between the RV entries and the signed plan. Initially, the software prompts the RV diagnosis and requests a confirmation of the appropriateness of the paper plan beam arrangement. The user is then presented with the RV prescription site, and is asked to ensure the proper organ is used to define the GTV, as well as the proper laterality (e.g. **left** parotid). The RV prescription modality is then reviewed vis‐à‐vis the corresponding plan parameters (e.g. energy, photons/electrons, MLC, wedges, bolus). The RV prescription fractionation regimen is then matched with the plan dose per fraction and number of fractions. The RV prescription isodose value is next correlated with the plan normalization scheme. Finally, the user is prompted to ensure that comments added to the prescription are properly implemented. A typical summary sheet is shown in Fig. 1(a).

The extracted RV parameters listed in Table 1 are then reviewed against the signed plan, to verify accuracy of transfer and sensibility. Parameters are divided into two categories. Constant (C) parameters, such as couch position, are not expected to vary between fields. The algorithm text compares such constant parameters to each other to ensure sameness. It was found that certain parameters could have a consistent value, yet be wrong (e.g. wrong tolerance used for all fields). To increase vigilance (denoted by a subscript v in Table 1), these parameters are entered manually once, and text‐compared with all entries. Unique (U) parameters, such as gantry angle, are expected to vary between fields. The user is prompted to manually review the parameter for each field. In addition, self‐consistency is reviewed among plan parameters by verifying that all fields have the same superior‐inferior jaw positions, and the posterior border of breast tangent fields is non‐divergent. Any deviation from expectation noted during the review will automatically generate a notification in the plan review summary sheet that must then be acknowledged by the checking physicist. This set of intra‐plan checks is by no means exhaustive, but the macro is sufficiently flexible so that each radiation oncology center could include additional tests.

**Table 1:** Each RV parameter listed above is reviewed against the signed plan. Constant (C) parameters are not expected to vary between fields. The software text compares these parameters to each other to ensure sameness. The v subscript denotes parameters entered once, and text‐compared with all entries. Unique (U) parameters are expected to vary between fields. The user is prompted to manually review each value.

*Parameter*	*Check type*	*Comments/additional checks*
Linac	$C_{v}$
Modality	C	Ensure proper modality (i.e., Photon vs electron)
Energy	$C_{v}$
Tolerance table	$C_{v}$
Field approval	C	Verify approval by dosimetrist
Dose Rate	$C_{v}$
Gantry angle	U
Collimator angle	$C_{v}$	Exception: Breast tangents
Lateral Jaws	U
Sup‐Inf Jaws	C
Couch positions	C
Couch angle	C	Used to alert user of non‐coplanar beams
Wedge	U
Bolus	U
Summed Dose per field	U	Difference between the prescription dose and the summed dose per field

### B. Inter‐plan Review

The inter‐plan review compares the current plan parameter to previous similar cases and identifies outlying plan parameters, potentially due to atypical circumstances (such as higher beam energy for obese patients) or due to errors. Once the intra‐plan self‐consistency review is completed, the user chooses the proper category of similar cases. In this work, similar cases are categorized according to diagnosis, anatomic site, laterality, delivery technique, and fractionation scheme, as described in Table 2. Field parameters displayed in Table 1 as well as dose, source‐skin distance (SSD), and number of control points per field are then imported into a spreadsheet containing parameters of previously reviewed similar cases. In order to have sufficient statistics, only the most commonly treated similar cases (shown in Table 2) were analyzed in this work.

**Table 2:** Categories of similar cases used in the inter‐plan review process. Grouping of parameters for similar case enables a meaningful parameter comparison. N denotes the number of cases analyzed in this work.

*Category*	*Organ*	*Position*	*Technique*	*Dose, Gy*	*Cone Down*	*N*
1	Prostate	Prone	IMRT	75.6	IMRT	24
2	Prostate	Supine	IMRT	45	seeds	5
3	Lt. Breast	Supine	Tangential Photons	46.8	Electrons	7
4	Rt. Breast	Supine	Tangential Photons	46.8	Electrons	9

The current plan data are compared with previous plans using SPC formalism, as previously described by Wheeler et al,^(2)^ and Pawlicki et al.^(3)^ In brief, SPC theory was originally developed for quality assurance of manufacturing processes. Each process parameter can be characterized by its location and dispersion, or average and range, as surrogates. The historical data of a specific plan parameter (such as the jaw position of a particular field) are $X_{1}$, $X_{2}$, …, $X_{L}$, $X_{\text{current}}$, where $X_{1}$ is the parameter value of the first plan analyzed, $X_{L}$ is the parameter value of the last plan analyzed, and $X_{\text{current}}$ is the parameter value of the plan being currently evaluated.

Generally, measurements are grouped into subgroups to avoid spurious readings. For a subgroup size n, the subgroup mean and range would be $\bar{X}=\frac{1}{n}\sum_{i=1}^{n}X_{i}$ and $R=\text{Max}(X_{1},X_{2},\ldots ,X_{n})-\text{Min}(X_{1},X_{2},\ldots ,X_{n})$. In this work, the objective is to evaluate a single plan, implying a subgroup size of unity. Therefore, the subgroup mean is simply the current value, $X_{\text{current}}$, and the subgroup range is defined as $R=|X_{Current}-X_{L}|$. The population average estimate can be approximated by the average of the subgroup averages, $\bar{\bar{X}}=\frac{1}{L}\sum_{i=1}^{L}X_{i}$ while the population range estimate can be approximated by the average subgroup range, $\bar{R}=\frac{1}{L}\sum_{i=1}^{L}\left|X_{i+1}-X_{i}\right|$.

In the SPC formalism, control charts plot each field parameter as a function of cases reviewed. Since the parameter is plotted in chronological order, the current plan parameter value appears as the last entry. Each parameter is described by its location and dispersion, yielding two control charts per parameter. Atypical control chart pair is shown in Figs. 1(c) and (d) for a breast patient's medial beam longitudinal couch coordinate. The 7th value in Fig. 1(c) represents the current plan parameter value being evaluated, while the 6th value in Fig. 1 (d) represents the current plan parameter value.

The SPC theory assumes a measurement representing a well controlled process will generally exhibit random fluctuation. When the process deviates from alignment, control charts can differentiate noise from potential signals using thresholds. The upper and lower mean control chart thresholds, configured to yield a 1% chance of false alarm (or 99% confidence in recognizing outliers), can be expressed as^(3)^

(1) $$A_{u,l}=\bar{\bar{X}}\pm t\cdot \frac{\bar{R}}{d_{2}\cdot \sqrt{n}}=\bar{\bar{X}}\pm 2.66\cdot \bar{R}$$

where t=3, and $d_{2}=1.128$. The upper and lower range control chart thresholds are

(2) $$R_{u,l}=\left(1\pm t\cdot \frac{d_{3}}{d_{2}}\right)\cdot \bar{R}$$

where $d_{3}=0.8525$, yielding $R_{u}=3.21\overset{ˉ}{R}$, and since R is calculated as absolute difference, $R_{l}=-1.21\cdot \overset{ˉ}{R}$ is set to 0.

Each current plan parameter is assessed by deriving the above quantities using previous plan parameters. Since each plan parameter is described by 2 control charts, the physicist would need to review hundreds of control charts per case, a burdensome task. Instead, the algorithm reviews each parameter against its upper and lower thresholds, and only creates flags and control charts for parameters exceeding tolerance. This helps the user to focus on exceeding values, but excludes reviewing trends in the data. The flags generated are documented in the plan review summary sheet.

## III. RESULTS

Forty‐five previously reviewed plans were evaluated using the algorithm. Generating the RV parameters report, transferring it to the spreadsheet, and performing the review takes about 2–4 minutes, depending on case complexity.

**Figure 2:**
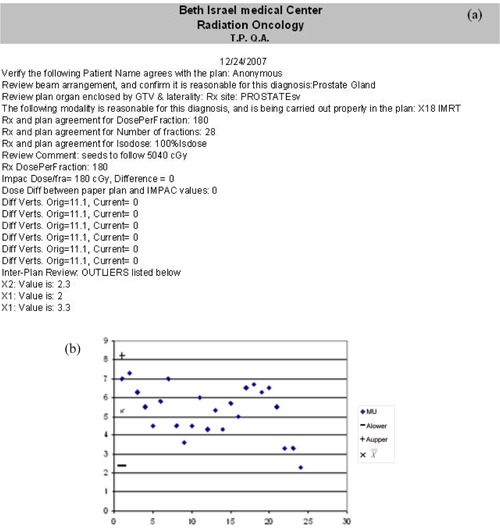
a plan review summary sheet for a prostate case exhibiting intra‐plan flags for inconsistent couch values, and inter‐plan flags for atypically low lateral jaw values of posterior fields. a representative control chart.

A typical summary printout containing three inter‐plan flags is shown in Fig. 1, with the corresponding control charts. The first two flags indicate the MU and couch longitudinal (Lng) values are substantially different than values of previous plans; the second Lng flag indicates the difference between this value and its predecessor exceeds an acceptable range. In Fig. 2, inconsistent couch positions were flagged by the intra‐plan review due to a clinical patient alignment that was only captured for the first field. In addition, the inter‐plan review flags unexpectedly narrow Jaws X1 and X1 values due to a change in rectal constraints.

Of the forty‐five previously reviewed cases, the algorithm also identified a prone prostate patient planned using a supine beam arrangement, and another involved unexpected gantry angles attributable to a hip prosthesis. There were also three improper tolerance tables, and summed field doses not adding to the prescription dose.

## IV. DISCUSSION

The formalism and its software implementation are useful in quickly identifying inconsistencies and highlighting differences between the reviewed plan and similar cases. This allows the reviewer to focus on ‘big picture’ items that should be carefully reviewed such as discrepancies between the prescription and plan, inappropriate application of technique to a specific clinical situation, or manual review of additional parameters not tracked by the RV system. However, this algorithm should only be clinically implemented after a facility's treatment protocols are carefully commissioned, so as to avoid lending credence to faulty processes.

As discussed above, the inter‐plan review process is expedited by presenting a list of outliers and their corresponding control charts, which helps the user to focus on exceeding values but precludes observing trends across the cases compared. The existence of such a trend, which amounts to a lack of statistical control,^(2)^ is periodically monitored by evaluating each mean parameter value against the corresponding control limits $A_{u}$, $A_{1}$. Control chart are displayed when a lack of statistical control is detected, as defined by:

a) One value falls outside ($A_{u}$ or Al), or
b) at least 2 out of 3 successive values fall on the same side of, and more than ($A_{u}$ or $A_{l}$) away from, the central line, or
c) at least 4 out of 5 successive values fall on the same side of, and more than ($A_{u}$ or $A_{l}$) away from, the central line, or
d) at least 8 successive values fall on the same side of the central line.

This analysis was repeated for each parameter range. This trend review was useful in detecting a shift in mean value, corresponding to a change in clinical protocol. For example, increasing the PTV margins expresses itself in larger jaw values, while placing stricter constraints on the bladder yields lower MU and dose/field in anterior fields. Data trends should be reviewed periodically and new patterns should be discussed with the staff. Moreover, when a protocol‐related change is noticed, the algorithm's statistics should be limited to correspond with the shift point, to avoid misidentifying errors.

## V. CONCLUSIONS

In this work, the pre‐treatment chart check process was compartmentalized into intra‐plan and inter‐plan reviews. The intra‐plan review is formalized as a series of computer‐guided tasks and self‐consistency macros that automatically document deviations. The inter‐plan review is based on binning similar plan parameters. The binned parameters enable experience‐quantification and facilitate automation. Forty‐five patient charts were analyzed using the software. The entire initial chart check process duration was reduced from about an hour to a few minutes. The code developed in this work allows the user to focus on the ‘forest’, trusting the software to track ‘trees’. This algorithm also allows multiple users (and potentially multiple centers) to share their experience.
